# *Toxoplasma* effector-induced ICAM-1 expression by infected dendritic cells potentiates transmigration across polarised endothelium

**DOI:** 10.3389/fimmu.2022.950914

**Published:** 2022-08-03

**Authors:** Emily C. Ross, Arne L. ten Hoeve, Jeroen P. J. Saeij, Antonio Barragan

**Affiliations:** ^1^ Department of Molecular Biosciences, The Wenner-Gren Institute, Stockholm University, Stockholm, Sweden; ^2^ Department of Pathology, Microbiology, and Immunology, University of California, Davis, Davis, CA, United States

**Keywords:** leukocyte, blood-brain barrier, apicomplexa, trans-endothelial migration, cell adhesion molecule (CAM), immune cell

## Abstract

The obligate intracellular parasite *Toxoplasma gondii* makes use of infected leukocytes for systemic dissemination. Yet, how infection impacts the processes of leukocyte diapedesis has remained unresolved. Here, we addressed the effects of *T. gondii* infection on the trans-endothelial migration (TEM) of dendritic cells (DCs) across polarised brain endothelial monolayers. We report that upregulated expression of leukocyte ICAM-1 is a feature of the enhanced TEM of parasitised DCs. The secreted parasite effector GRA15 induced an elevated expression of ICAM-1 in infected DCs that was associated with enhanced cell adhesion and TEM. Consequently, gene silencing of *Icam-1* in primary DCs or deletion of parasite GRA15 reduced TEM. Further, the parasite effector TgWIP, which impacts the regulation of host actin dynamics, facilitated TEM across polarised endothelium. The data highlight that the concerted action of the secreted effectors GRA15 and TgWIP modulate the leukocyte-endothelial interactions of TEM in a parasite genotype-related fashion to promote dissemination. In addition to the canonical roles of endothelial ICAM-1, this study identifies a previously unappreciated role for leukocyte ICAM-1 in infection-related TEM.

## Introduction

The blood-brain barrier (BBB) separates the blood from the brain parenchyma. In the neurovascular unit, the endothelium regulates and heavily restricts the movement of molecules, cells and pathogens between the two compartments (1). Yet, leukocytes can enter the brain parenchyma in a tightly regulated fashion, which is crucial to resolve infection or other insults. Paradoxically, leukocytes can also be utilized by pathogens to gain access to the brain (2).

The process of leukocyte trans-endothelial migration (TEM), also termed diapedesis, entails signalling cascades (3) and a tight interplay of leukocytes with the endothelium (4). In the leukocyte´s approach to the site of TEM, crucial interactions between intercellular cell adhesion molecule-1 (ICAM-1) on the endothelium and leukocyte integrins, such as LFA-1 (α_L_β_2_; CD11a/CD18), precede translocation (5, 6). However, while most leukocyte subsets express ICAM-1, the putative role of leukocyte ICAM-1 in diapedesis has remained unresolved (7, 8). Intracellular microbes hijack host cell functions and, therefore, the study of host-pathogen interactions can provide unique cues on the biology of leukocyte migration.

The obligate intracellular parasite *Toxoplasma gondii* chronically infects a significant portion of the global human population and warm-blooded vertebrates (9). Reactivated infection in immunocompromised individuals or congenital infection in the developing foetus can result in severe neurological manifestations (10). Yet, primary infection is often asymptomatic or accompanied by mild flu-like symptomatology (10). This implies that early passage to the central nervous system is generally clinically silent and yields chronic, possibly life-long, latent infection (11, 12). In Europe and North America, three clonal lineages of *T. gondii* prevail (type I, II, III), with type II strains being commonly carried by humans and by animals used for meat consumption (13, 14).

*T. gondii* actively infects nucleated cells, including immune cells. Parasitised dendritic cells (DCs) and other mononuclear phagocytes mediate systemic dissemination of *T. gondii* (15, 16). Passage of *T. gondii* to the brain parenchyma is restricted by the BBB (17) and alternative translocation pathways, including trafficking inside leukocytes, have been proposed (12, 18). Secretory rhoptry organelles are discharged upon host cell invasion and once the parasite resides in its intracellular niche, the parasitophorous vacuole (PV), dense granules contents are discharged. Notably, effector proteins from these two secretory compartments impact host cell transcription and signalling, and thus the host-parasite interaction (19, 20). The effector protein TgWIP is secreted from rhoptries into the host cytosol and impacts the migration of parasitised DCs across transwell filters (21). In addition, a number of dense granule proteins (GRA) are transported across the PV into the host cell cytosol, which is dependent on Myc regulation proteins (MYR). In contrast, the effector functions of the polymorphic effector protein GRA15, which is inserted into the PV membrane, do not depend on MYR (22). GRA15 from type II *T. gondii* strains activates the NFκB pathway, leading to macrophage production of inflammatory cytokines (23).

We recently described an important role for leukocyte integrins and endothelial CAMs in the transmigration of *T. gondii*-challenged DCs across polarised monolayers of primary murine brain endothelium (24). However, while endothelial ICAM-1 is crucial for TEM (4), the implication of leukocyte ICAM-1 in diapedesis and, specifically, in TEM associated with microbial pathogenesis has remained unexplored (25). Here, we describe a role for *T. gondii* effector-induced leukocyte ICAM-1 in TEM and provide a framework for further exploration of the described differences between *T. gondii* lineages in transmigration *in vitro* and systemic dissemination in mice (26).

## Materials and methods

### Ethics statement

The Regional Animal Research Ethical Board, Stockholm, Sweden, approved protocols involving extraction of cells from mice, following proceedings described in EU legislation (Council Directive 2010/63/EU).

### Parasite culture and cell lines

*T. gondii* type I (RH) and II (ME49, PRU) strains and mutant lines (ΔMYR1, ΔTgWIP, ΔGRA15) are detailed in **Supplementary Table 1** and were maintained by serial 48 h passaging in human foreskin fibroblasts (HFF; CRL-2088, American Type Culture Collection). HFF and mouse brain endothelial cells (bEnd.3, CRL-2299, American Type Culture Collection) were cultured in Dulbecco’s modified Eagle’s medium (DMEM, ThermoFisher scientific) with 10% heat inactivated foetal bovine serum (FBS, Sigma), gentamicin (20 μg/ml, Gibco), L-glutamine (2 mM, Gibco) and HEPES (10 mM, Gibco). Cell cultures and parasites were grown in a humidified atmosphere containing 5% CO2 at 37°C and regularly tested for *Mycoplasma*.

### Primary DCs

Murine bone marrow-derived DCs were generated as previously described (27). Briefly, cells from bone marrow of 6-10 week old male or female C57BL/6NCrl mice (Charles River) were cultivated in RPMI 1640 with 10% FBS, gentamicin (20 μg/ml), glutamine (2mM) and HEPES (0.01 M), referred to as complete medium (CM), and supplemented with 10 ng/ml recombinant mouse GM-CSF (Peprotech). Loosely adherent cells (DCs) were harvested after 6 or 8 days of maturation.

### Polarisation parameters: Permeability assay and transendothelial electrical resistance (TEER)

bEnd.3 cells were cultured to 80% confluence then seeded onto transwells (8 µm pore size; BD Biosciences) and grown for 5 d until they reached polarisation, as defined below. For evaluation of cell monolayer permeability following transmigration, FITC-dextran (3 kDa; Life tech) was added to the upper compartment of the transwell at a concentration of 12.5 μg/ml for 90 min. Medium was collected from the lower compartment, and fluorescence was measured in a fluorometer (EnSpire Multimode Plate Reader, Perkin Elmer) at 485 nm excitation 520 nm emission. Fluorescence intensity was measured as arbitrary fluorescence units (a.u.) at a sensitivity of < 1 fmol fluorescein/well, as specified by the manufacturer. Polarised monolayers were defined by a TEER ≥ 250 Ω * cm^2^, as measured using an Ohmmeter (Millipore, Bedford, MA) and correcting measurements with the formula: Unit Area Resistance (TEER) = Resistance (Ω) * Effective Membrane Area (cm^2^). TEER was measured before and after transmigration or treatments. Values are shown as percentage (%) of TEER related to TEER prior to treatments or transmigration.

### Motility and transmigration assays

Motility assays were performed as previously described (24). Briefly, DCs were challenged with freshly egressed *T. gondii* tachyzoites (MOI 3) for 4 h (resulting in 70–80% infection frequency) and with soluble reagents as indicated. DCs were then added to 96-well plates pre-cultured with bEnd.3 cells. Live cell imaging was performed for 1 h, 1 frame/min, at 10X magnification (Z1 Observer with Zen 2 Blue v. 4.0.3, Zeiss). Time-lapse images were consolidated into stacks and motility data was obtained from 30 cells/condition (Manual Tracking, ImageJ) yielding mean velocities (Chemotaxis and migration tool, v. 2.0). Infected cells were defined by GFP^+^ or RFP^+^ cells, as indicated.

Transmigration assays were performed as previously described (24). Briefly, DCs were cultured with CM ± freshly egressed *T. gondii* tachyzoites (MOI 2, 4 h) and then transferred to transwells with pre-cultured bEnd.3 monolayers. After 16 h, transmigrated DCs were put on ice for 1 h to disassociate adherent cells, and then counted manually in a Bürker chamber by light microscopy. For each independent experiment, duplicate or triplicate technical replicates were assessed and means calculated.

### Motility assays under shear stress

Flow motility assays were performed as previously described (24). Briefly, DCs were challenged as stated under motility assay and then added to fluidic channels (μ-Slide VI0.4; Ibidi) with confluent bEnd.3 cell monolayers and allowed to adhere for 10 min. Phase-contrast and fluorescence images were first captured in static condition. Fluidic shear stress was then applied by flowing CM at 0.2 dyn/cm^2^ through the channels. Live cell imaging was immediately initiated and images acquired every 10 s for up to 10 min, at 10X magnification (Z1 Observer with Zen 2 Blue v. 4.0.3, Zeiss). Time-lapse images were consolidated into stacks and motility and path-length data obtained from 20-30 cells (Manual Tracking, ImageJ) yielding individual cell velocities and pathlengths (Chemotaxis and migration tool, v 0.2.0).

### Flow cytometry

Bone marrow-derived DCs were cultured in CM ± freshly egressed *T. gondii* tachyzoites (MOI 1) for 24 h. Cells were stained on ice in FACS buffer (1% FBS and 1 mM EDTA in PBS) with Live/Dead Violet (L34955, Life technologies), anti-mouse CD11c PE-Cy7 (clone N418, 25-0144-82, eBioscience) and anti-mouse CD18 PE (clone M18/2, 101407, BioLegend) or anti-mouse CD29 Alexa Fluor 647 (clone HMβ1-1, 102213, BioLegend) or anti-mouse CD54 APC (clone YN1/1.7.4, 116119, BioLegend) or IgG2a PE isotype control (clone R35-95, 553930, BD Pharmingen) antibodies, fixed with 2% PFA and acquired on a BD LSRFortessa flow cytometer (BD Biosciences). The results were then analysed with FlowJo software (v. 10, FlowJo LLC). Prior to staining, cells were blocked in FACS buffer supplemented with the Fc blocking anti-mouse CD16/CD32 antibody (clone 93, 14-0161-82, eBioscience). Infected cells were defined as GFP^+^ cells when challenged with GFP-expressing *T. gondii*. For *T. gondii* strains that lack a fluorescent reporter (PRUΔku80 and PRUΔMYR1, **Supplementary Figure 1D**), cells were permeabilized with 1% Triton X-100 in FACS buffer and stained with primary mouse anti-*T. gondii* SAG1 (clone P30/3, MA1-83499, ThermoFisher) and chicken anti-mouse IgG (H+L) Alexa Fluor 488-conjugated (A-21200, ThermoFisher) secondary antibodies. Infected cells were then defined as Alexa Fluor 488^+^.

### Immunostainings

Unchallenged or *T. gondii*-challenged DCs were plated on coverslips pre-coated with 1% gelatin (BioRad). Cells were fixed (4% PFA, Sigma-Aldrich), blocked (5% FBS in PBS for 2 h), then incubated with rat anti-mouse CD54 (ICAM-1; clone YN1/1.7.4; eBioscience) at 1:200 overnight at 4°C. Cells were then stained with chicken anti-rat IgG (H+L) Alexa Fluor 594-conjugated secondary antibody (1:1000) and DAPI for 2 h, mounted and imaged using a 63X objective (DMi8, Leica Microsystems). The ICAM-1 signal intensity of *T. gondii*-infected cells (GFP^+^) and by-stander cells (GFP^-^) from randomly chosen fields of view (4-5/per condition and experiment) was measured in ImageJ, and corrected for with the following formula; Integrated Density – (Area of selected cell X Mean fluorescence of background readings). The corrected value is shown in graphs as arbitrary units (a.u.). For live imaging, *T. gondii*-challenged DCs were blocked with anti-CD16/CD32 antibody (clone 93, 14-0161-82, eBioscience), stained with anti-mouse CD54-APC (clone YN1/1.7.4, 116119, BioLegend) antibody on ice, washed, seeded on bEnd.3 cells and imaged for 1 h.

### Lentiviral vector production and *in vitro* transduction

A self-complementary hairpin DNA oligo targeting *Icam1* (shICAM1, TRCN0000218257, Genscript) mRNA was on self-inactivating lentiviral vectors (pLL3.7) with eGFP reporter expression (**Supplementary Table 2**). Transfer plasmid (shRNA targeting Icam1 or Luc) was co-transfected with psPAX2 (12260, Addgene) packaging vector and pCMV-VSVg (8454, Addgene) envelope vector into Lenti-X 293 T cells (Clontech) using Lipofectamine 2000 (Invitrogen). The resulting supernatant was harvested 24 h and 48 h post-transfection. Supernatants were centrifuged to eliminate cell debris and filtered through 0.45-mm cellulose acetate filters. DCs (5 days post-bone marrow extraction) were transduced by adding lentiviral supernatants in the presence of DEAE dextran (8 µg/ml; Sigma-Aldrich) to cells for 4 h. After 2-3 days (7-8 days post-bone marrow extraction), transduction efficiency was examined for eGFP expression by epifluorescence microscopy (Z1 Observer with Zen 2 Blue v. 4.0.3, Zeiss) followed by expression analysis by qPCR for knock-down of targeted mRNA.

### Polymerase chain reaction (PCR)

Total RNA was extracted using TRIzol reagent (Sigma-Aldrich). First-strand cDNA was synthesised with Maxima H Minus Reverse Transcriptase (Thermo Fisher). Real-time quantitative polymerase chain reaction (qPCR) was performed using SYBR green PCR master mix (Kapa biosystems), forward and reverse primers (200 nM), and cDNA (100 ng) with a QuantStudio™ 5 real-time PCR system (ThermoFisher). Glyceraldehyde 3-phosphate dehydrogenase (GAPDH) was used as a house-keeping gene to generate ΔCt values; 2−ΔCt values were used to calculate relative expression. All primers (Invitrogen) were designed using the Get-prime or Primer-BLAST software (**Supplementary Table 3**).

### Statistical analyses

All statistics were performed with Prism software (v. 8, GraphPad). Multiple comparisons were carried out by one-way ANOVA, Sidak’s *post-hoc* test, while 2-sample tests were conducted with a Student’s t-test on normally distributed sample populations. A *p*-value less than 0.05 was considered to be statistically significant. All data are from 3 or more biological replicates separated in time.

## Results

### Parasite effectors impact the transmigration frequency of parasitised DCs across polarised endothelial cell monolayers in a genotype-related fashion

**Figure 1:**
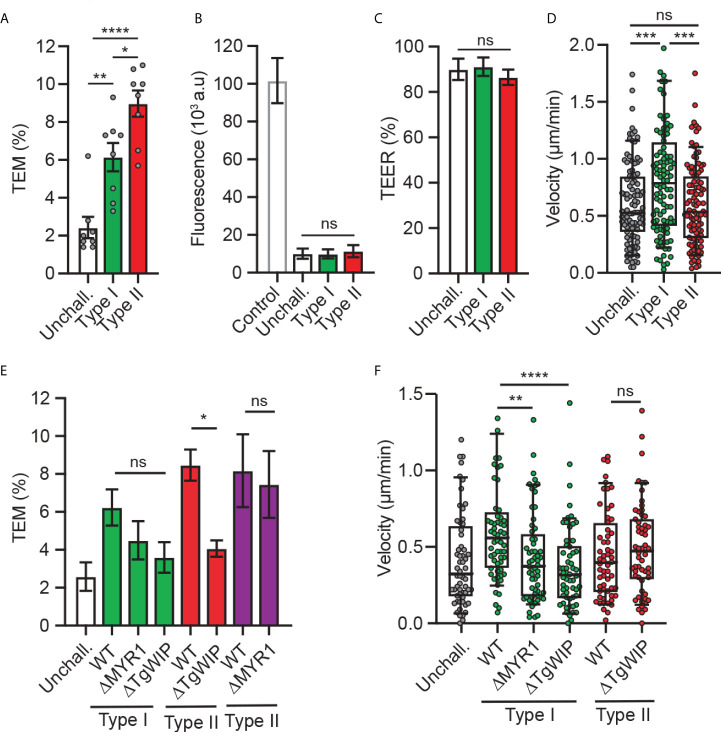
Transmigration and motility of *T. gondii*-challenged DCs on bEnd.3 monolayers. **(A)** Transendothelial migration (TEM) frequency of unchallenged or *T. gondii*-challenged (type I or II) DCs across bEnd.3 cell monolayers shown as percentage (%) of DCs added in the upper well. **(B)** Permeability of bEnd.3 monolayers to FITC-dextran (3 kDa) after DC TEM. Data are shown as arbitrary fluorescence units (a.u.) as specified under materials and methods. **(C)** TEER values of bEnd.3 cells relative to TEER values at initiation of the assay (100%). **(D)** Box-and-whisker and scattered dot plots represent median velocities (μm/min) of unchallenged and *T. gondii*-challenged DCs on bEnd.3 monolayers. Each dot represents one tracked cell (n = 100). **(E)** TEM frequencies of unchallenged DCs or DCs challenged with type I (RH; green bars) or type II (ME49-PTG; red bars, PRU; purple bars) WT or mutant strains across bEnd.3 cell monolayers shown as percentage (%) of DCs added in the upper well. **(F)** Box-and-whisker and scattered dot plots represent median velocities (μm/min) of unchallenged and *T. gondii*-challenged DCs on bEnd.3 monolayers. Each dot represents one tracked cell (n = 60). Bar graphs represent the mean ± s.e.m of 3-4 independent experiments. *P < 0.05, **P < 0.01, *** *P* < 0.001, **** *P* < 0.0001, ns: non-significant, by one-way ANOVA, Sidak’s *post-hoc* test.

Previous studies established that *T. gondii*-infected DCs perform TEM across polarised endothelial cell monolayers at a higher frequency than unchallenged DCs (24) and that the shuttling function of DCs in mice is related to the parasite genotype (26). To assess whether TEM is genotype-dependent, DCs infected with a type I prototypic strain (RH) or type II strain (ME49) were assessed for transmigration across polarised endothelium (bEnd.3). While both genotypes induced TEM, the type II strain consistently induced superior transmigration frequencies (**Figure 1A**), at comparable infection frequencies **(** **Supplementary Figure 1A**). As previously described (24), transmigration of *T. gondii*-challenged DCs occurred in the absence of elevated permeability to the low-molecular weight tracer FITC-dextran and with maintained TEER (**Figures 1B, C** **)**, indicating preserved cellular barrier integrity.

Because rolling, arrest and crawling are steps that precede the transmigration of leukocytes across endothelium (28), next, we assessed the migratory behaviour of infected DCs on endothelium. Interestingly, while type II-infected DCs exhibited superior transmigration frequency compared to type I-infected DCs (**Figure 1A**), type I-infected DCs, but not type II-infected DCs, exhibited an elevated motility on endothelium (**Figure 1D**). These relative but consistent strain-related phenotypical differences motivated an exploration of strain-related effectors. First, we confirmed that the rhoptry effector TgWIP impacted transmigration for type II-infected DCs (21). However, because the elevation of transmigration was only partially reduced upon TgWIP deletion (**Figure 1E**), we hypothesized that additional secretory pathways were in play. Challenge with parasite mutants (type I and II) with a deletion of MYR1, which blocks secretion of GRAs beyond the PV, yielded a non-significant impact on transmigration frequencies (**Figure 1E**; **Supplementary Figures 1B, C** **)**. TgWIP and MYR1 deletion in type I parasites decreased DC motility on endothelium, in contrast with TgWIP deletion in type II parasites **(** **Figure 1F** **)**. We conclude that parasite genotype-related differences exist in the migratory activation of infected DCs and that both rhoptry-related secretion (TgWIP) and non-MYR1-associated effector(s) impact transmigration.

### ICAM-1 and integrin expression in *T. gondii*-challenged DCs

**Figure 2:**
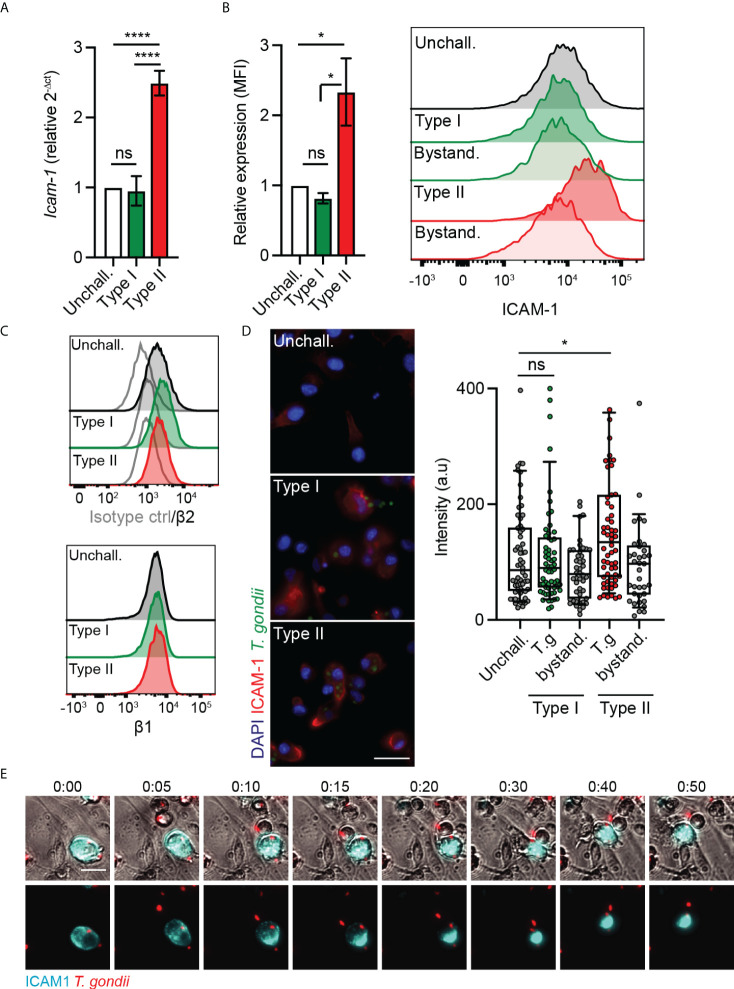
Parasite genotype-dependent upregulation and expression of ICAM-1 in DCs. **(A)** *Icam-1* mRNA expression (2^-ΔCt^) in DCs challenged with type I (RH) or II (ME49-PTG) *T. gondii* tachyzoites for 4 h, relative to unchallenged condition. **(B)** Bar graphs show relative expression of ICAM-1 (CD54) at 24 h post-challenge of CD11c^+^ cells with *T. gondii* tachyzoites (GFP-expressing type I (RH) or type II (ME49-PTG), MOI 1), assessed by flow cytometry. Mean fluorescence intensity (MFI) was related to that of unchallenged CD11c^+^ in complete medium (CM, normalised to 1). Histogram shows fluorescence intensity distributions for indicated cell populations. **(C)** Histograms show fluorescence intensity distributions of β2, β1 integrin and isotype antibody control for indicated cell populations, respectively, and are representative of 3 independent experiments. **(D)** Micrographs represent DCs infected with GFP-expressing *T. gondii* type I (RH) or II (ME49-PTG) (green) and stained for ICAM-1 (red) and nuclei (blue). Box-and-whisker and scattered dot plots show ICAM-1 staining intensity of DCs. Each dot represents the corrected intensity fluorescence of one cell (n = 40-62), as detailed in materials and methods. **(E)** Representative micrographs of live cell imaging of type II (ME49) *T. gondii*-challenged DCs stained with anti-ICAM-1 APC antibody, on bEnd.3 cell monolayers. Scale bar = 20 µm. Bar graphs represent the mean ± s.e.m of 3-4 independent experiments. *P < 0.05, **** *P* < 0.0001, ns: non-significant, by one-way ANOVA, Sidak’s *post-hoc* test.

ICAM-1 and integrins were previously reported to play a role in the migratory phenotypes of *T. gondii*-infected DCs and monocytes, with an impact of antibody-blockade on TEM (24, 29). However, because ICAM-1 and integrins are expressed by both the endothelium and leukocytes (30), their precise roles in TEM have remained unresolved. We therefore performed a detailed analysis of ICAM-1 and integrin expression in *T. gondii*-challenged DCs. We found that challenge of DCs with type II parasites induced elevated ICAM-1 transcription (**Figure 2A**). Consistently, flow cytometry analyses showed elevated ICAM-1 expression in type II-infected DCs but not in challenged non-infected DCs (**Figure 2B**), indicating absence of a by-stander effect. In contrast, β1 and β2 integrin expression remained essentially unchanged (**Figure 2C**), as previously described (24). Infection with type I parasites non-significantly impacted ICAM-1 expression in DCs (**Figures 2A, B** **)**. Immunofluorescence intensity analyses corroborated elevated ICAM-1 signal in type II-infected DCs in absence of elevated signal in non-infected by-stander DCs or in type I-infected DCs (**Figure 2D**). Jointly, this indicated effects linked to parasite infection and to parasite genotype. Moreover, in live cell assays, a redistribution of the ICAM-1 fluorescence signal to the trailing edge of infected DCs preceded and accompanied their migration on endothelial cell monolayers (**Figure 2E**). We conclude that challenge of DCs with type II *T. gondii* led to a higher expression of ICAM-1, with a redistribution of ICAM-1 upon migration of parasitised DCs on endothelium.

### Leukocyte ICAM-1 is implicated in the motility on endothelium and TEM of infected DCs in a parasite genotype-related fashion

**Figure 3:**
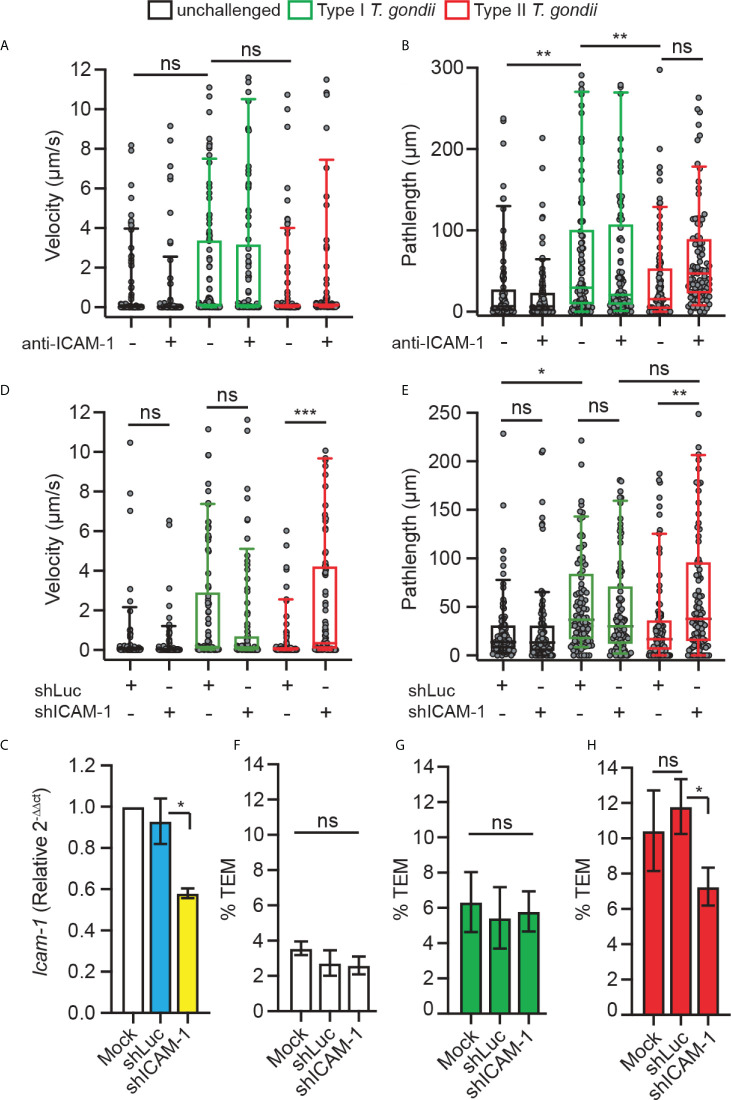
Implication of DC ICAM-1 in motility and TEM. **(A, B)** Box-and-whisker and scattered dot plots represent median velocities (μm/s) and pathlengths (μm), respectively, of type I (RH) or type II (ME49)-challenged DCs on bEnd.3 ± antibodies. Grey circles represent velocities or pathlengths, respectively, from individual cells. 95-100 cells were tracked per condition, from 3 independent experiments. **(C)** *Icam-1* mRNA expression (2^-ΔΔCt^) in DCs transduced with shICAM-1 or control shLuc lentivirus relative to mock-treated cells (mock set to 1.0). **(D, E)** Box-and-whisker and scattered dot plots represent median velocities (μm/s) and pathlengths (μm), respectively, of mock-treated DCs and DCs transduced with lentiviral vectors targeting *Icam-1* mRNA (shICAM-1) or a non-expressed target (shLuc) challenged with type I (RH) or II (ME49) *T. gondii* on bEnd.3. Grey circles represent velocities or pathlengths, respectively, from individual cells. 95-100 cells were tracked per condition, from 3 independent experiments. **(F–H)** TEM frequencies of unchallenged DCs **(F)**, type I (RH)-challenged DCs **(G)** and type II (ME49)-challenged DCs **(H)** across bEnd.3 cell monolayers shown as percentage (%) of DCs added in the upper well. Bar graphs represent the mean ± s.e.m of 3-4 independent experiments. *P < 0.05, **P < 0.01, ***P < 0.001, ns: non-significant, by one-way ANOVA, Sidak’s *post-hoc* test.

To address the function of DC ICAM-1 in the motility and transmigration of parasitised DCs, we applied combined approaches. First, based on previous findings indicating an implication of CAMs in TEM under static conditions (24), we assessed the velocities and pathlengths of *T. gondii-*infected DCs on bEnd.3 monolayers under shear stress. We found that type I-challenged DCs had a tendency for elevated velocities and significantly elongated pathlengths compared with type II-challenged DCs or unchallenged DCs, in presence or absence of antibody blockade using ICAM-1 mAb (**Figures 3A, B** **)**. However, one of several caveats of antibody blockade is that it presumably targets both endothelial and leukocyte ICAM-1. Next, to specifically assess the role of leukocyte ICAM-1, we gene silenced *Icam-1* in DCs. Cell suspensions with transduced primary DCs (30-40% eGFP^+^, **Supplementary Figure 2**) presented a reduction in *Icam-1* mRNA expression related to control shLuc-transduced DCs (**Figure 3C**). Importantly, *Icam-1* silencing significantly elevated velocities and pathlengths in type II-challenged DCs but not type I-challenged DCs (**Figures 3D, E** **)**. Conversely, while transmigration frequencies of unchallenged and type I-challenged DCs where non-significantly affected **(** **Figures 3F, G** **),** the transmigration frequencies of type II-challenged DCs across endothelial monolayers were significantly decreased in shICAM-1-transduced DCs compared with mock- or shLuc-transduced DCs **(** **Figure 3H** **).** We conclude that gene silencing of ICAM-1 (*Icam-1*) significantly inhibits transmigration of type II-challenged DCs but not of type I-challenged DCs. Consistent with motility data under static conditions (**Figure 1**), the inferior velocities and pathlengths under shear stress by type II-challenged DCs are indicative of ICAM-1-dependent adherence and TEM, while these effects are minimal or non-significant in type I-challenged DCs.

### The parasite effector GRA15 impacts ICAM-1 expression, motility and TEM of parasitised DCs

**Figure 4:**
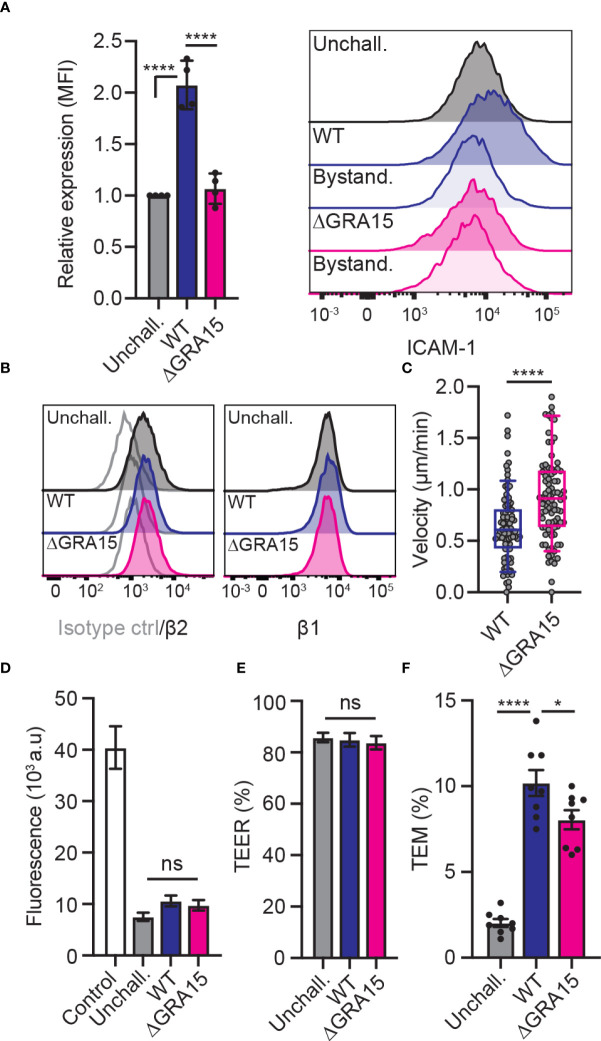
Impact of the effector GRA15 on ICAM-1 expression, motility and TEM of DCs. **(A)** Relative expression of ICAM-1 (CD54) at 24 h post-challenge of CD11c^+^ cells with *T. gondii* tachyzoites (GFP-expressing PRU WT or PRUΔGRA15, MOI 1), assessed by flow cytometry. Mean fluorescence intensity (MFI) was related to that of unchallenged CD11c^+^ in complete medium (CM, normalised to 1). Histogram shows fluorescence intensity distributions for indicated cell populations. **(B)** Histograms show fluorescence intensity distributions of β2, β1 integrin and isotype antibody control for indicated cell populations, respectively, and are representative of 3 independent experiments. **(C)** Box-and-whisker and scattered dot plots represent median velocities of WT-challenged or ΔGRA15-challenged DCs (μm/min). Grey circles represent velocities from individual cells. 90 cells were tracked per condition, from 3 independent experiments. **(D)** Permeability of bEnd.3 cell monolayers to FITC-dextran (3 kDa) following TEM of unchallenged, WT-challenged or ΔGRA15-challenged DCs. **(E)** TEER values of bEnd.3 cells relative to TEER values at initiation of the assay (100%). **(F)** TEM frequencies of unchallenged, WT-challenged or ΔGRA15-challenged DCs across bEnd.3 cell monolayers shown as percentage (%) of DCs added in the upper well. Bar graphs represent the mean ± s.e.m of 4 independent experiments. *P < 0.05, ****P < 0.0001, ns: non-significant, by one-way ANOVA followed by Sidak’s *post-hoc* test **(A, D, E, F)** or Student’s *t*-test **(C)**.

The findings that ICAM-1 silencing impacted transmigration in type II-infected DCs but not in type I-infected DCs motivated a search for putative effectors. Consistent with our phenotypical data, the dense granule protein GRA15 was suspected because it is inactive in the type I RH strain but active in type II strains (31), and its effects do not depend on the MYR translocon (23). To test whether GRA15 is involved in the type II-induced increased expression of ICAM-1, DCs were challenged with wild-type (WT) or GRA15-deficient (ΔGRA15) *T. gondii* lines and the expression of ICAM-1 was assessed by flow cytometry. Consistent with previous findings (**Figure 2**), WT-challenged DCs elevated ICAM-1 expression compared with unchallenged DCs **(** **Figure 4A**). In sharp contrast, DCs challenged with ΔGRA15 failed to elevate ICAM-1 expression **(** **Figure 4A** **)**, while integrin expression was maintained (**Figure 4B**). Importantly, DCs challenged with ΔGRA15 parasites presented elevated motility on endothelium (**Figure 4C**) and, conversely, reduced transmigration frequencies across polarised endothelium **(** **Figures 4D–F** **)**. We conclude that the increased expression of ICAM-1 in *T. gondii* type II-infected DCs is GRA15-dependent. Jointly, the data show that GRA15 expression is associated with elevated ICAM-1 expression, reduced velocities on endothelium and elevated TEM frequencies.

## Discussion

The mechanisms of leukocyte diapedesis across endothelium, and specifically DC trafficking across the BBB, entail complex signalling cascades that classically involve endothelial ICAM-1 (32). The upregulated expression of leukocyte ICAM-1 in *T. gondii*-infected DCs, in the absence of a by-stander effect, motivated an assessment of putative roles in TEM.

**Figure 5:**
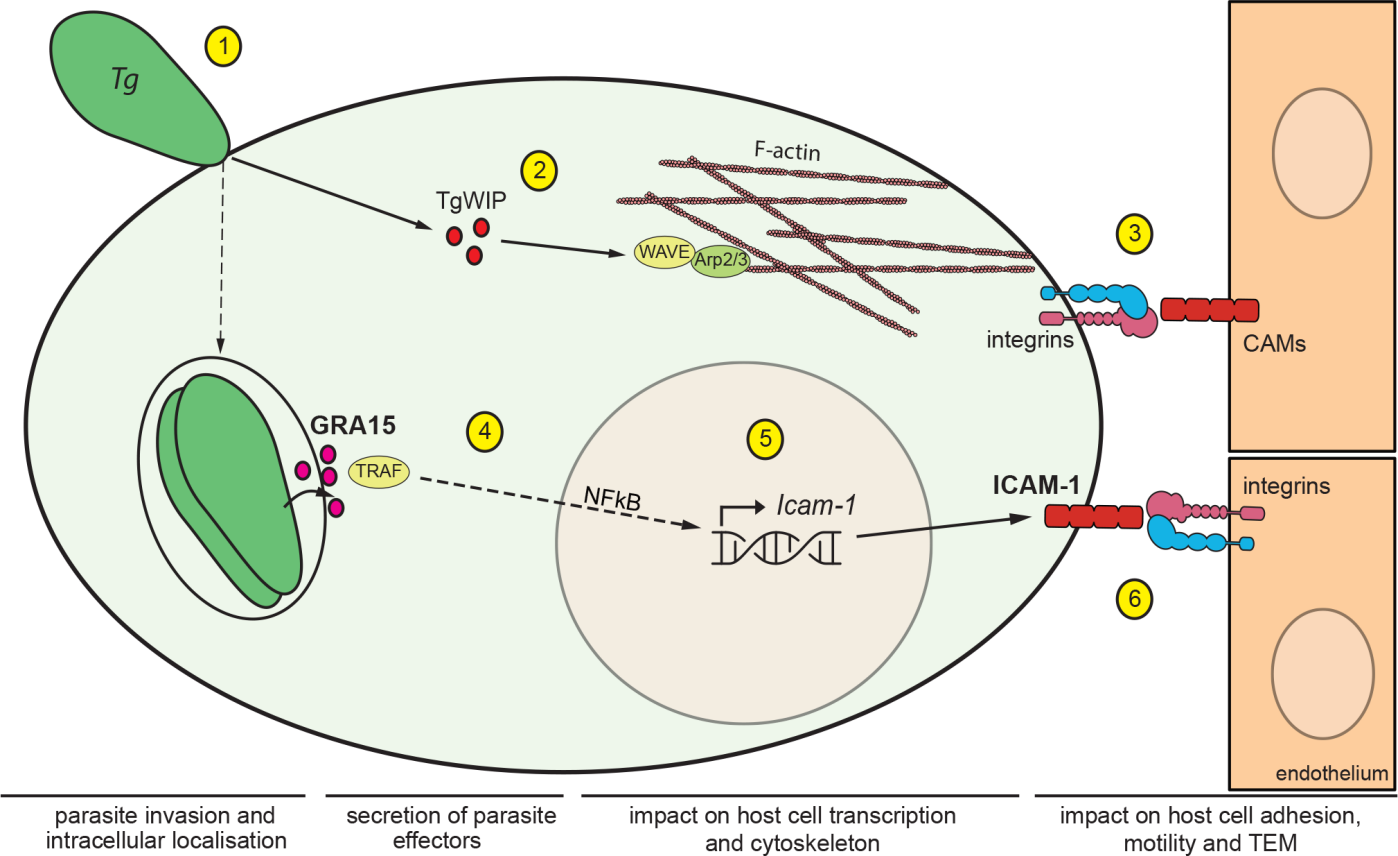
Impact of *T. gondii* secreted effectors on ICAM-1 expression and TEM of DCs. Schematic illustration of the signalling pathways mediating migratory activation and TEM of *T. gondii*-infected DCs across polarised endothelium. **(1)** *T. gondii* (*Tg*) actively invades leukocytes by its own actinomyosin-driven gliding motility and replicates intracellularly in the non-fusogenic parasitophorous vacuole (PV) surrounded by the PV membrane. **(2)** Upon invasion, the rhoptry secretory organelles are discharged into the host cell cytosol. The secreted effector TgWIP interacts with the host WAVE complex, which regulates filamentous (F)-actin dynamics *via* the Arp2/3 complex and drives enhanced cell motility (21). **(3)** In hypermigratory infected DCs, interactions between leukocyte β1/β2 integrins and endothelial cell adhesion molecules, including ICAM-1, regulate the TEM of DCs. Notably, infected DCs transit from integrin-independent amoeboid motility in extracellular matrix to integrin-dependent migration on endothelium. This motility mode switch facilitates TEM (24). **(4)** Following invasion, *T. gondii* replicates within the formed PV and secretes the dense granule protein GRA15, which is exported to the host cytosolic side of the PV membrane. GRA15 interacts with TNF receptor associated factors (TRAFs) that mediate translocation of the transcription factor NFκB to the host cell nucleus (23). **(5)** Nuclear NFκB activates transcription of *Icam-1* in the host cell nucleus with elevated mRNA expression in infected DCs, GRA15-dependently and in the absence of a by-stander effect. **(6)** Elevated expression of ICAM-1 at the DC membrane enhances cell adhesion, reduces cell motility and elevates TEM frequency *via* interactions with endothelial integrin moieties.

We demonstrate a role for leukocyte ICAM-1 in the motility on endothelium and the transmigration across polarised cerebral endothelium of DCs infected with the predominant type II *T. gondii* strains. Importantly, transcriptional upregulation of *Icam-1* was accompanied by elevated expression of ICAM-1 in infected DCs but not in by-stander DCs, indicating direct effects of intracellular parasitisation. Moreover, cell adhesion, motility and TEM *in vitro* were modulated by DC ICAM-1 expression. Of note, *T. gondii* infection also upregulates ICAM-1 expression in endothelial cell monolayers and the brain microvasculature (17, 33, 34). Therefore, antibody-blockade studies have correctly assumed an inhibitory effect on endothelial ICAM-1 (24, 29, 35) and the putative role of leukocyte ICAM-1 has remained unaddressed. Thus, in addition to endothelial ICAM-1, the present findings reveal that leukocyte ICAM-1 is implicated in TEM of parasitised DCs (**Figure 5**).

We recently reported a role for leukocyte β1/2 integrins in the TEM of parasitised DCs (24). A major binding partner for ICAM-1 is the β2 integrin LFA-1 (5, 6) and microvascular endothelium also expresses integrins, including LFA-1 (36). Thus, both integrins and ICAM-1 on leukocytes may interact with their corresponding moieties on endothelium, creating integrin-CAM interactions and, reciprocally, CAM-integrin interactions. The present study identifies leukocyte ICAM-1 as an additional determinant of DC transmigration *in vitro*. Along these lines, a critical role for ICAM-1 in mature DCs has been demonstrated for long-lasting contacts with CD8^+^ T cells at the ‘immunological synapse’ (25). In contrast, ICAM-1 expression by neutrophils has been associated with phagocytosis and reactive oxygen species (ROS) responses but was not essential for transmigration (37). We provide proof-of-concept that leukocyte ICAM-1 can contribute to the TEM of DCs in the context of infection. Further studies need to address the contribution of ICAM-1 in other leukocytes, other inflammatory conditions and *in vivo* (7, 38).

The data suggest an implication of leukocyte ICAM-1 in the pathogenesis of toxoplasmosis. Interestingly, recent work reported that ICAM-1 is elevated in the cerebral microvasculature of mice challenged with *T. gondii* (17). Endothelial and epithelial ICAM-1 play pivotal roles in microbial pathogenesis, including the sequestration of *P. falciparum*-infected erythrocytes to endothelium in cerebral malaria (39), viral binding to epithelium (40) and bacterial invasion (41). Similarly, soluble ICAM-1 and ICAM-1 antibodies reduced the transmigration of extracellular *T. gondii* tachyzoites across polarized epithelial monolayers (42). Thus, while the roles of endothelial and epithelial ICAM-1 in diapedesis are well established, the functions of leukocyte ICAM-1 have remained unelucidated in relation to microbial pathogenesis and diapedesis, including ICAM-1 isoform expression and function (43). Hypothetically, because the elevated expression of ICAM-1 in *T. gondii*-infected DCs is associated with endothelial adhesion, leukocyte ICAM-1 may contribute to mechano-transduction and therefore to TEM, as shown for endothelial ICAM-1 (44). Diapedesis encompasses tightly regulated cellular processes and signalling cascades. Thus, to promote its transportation in leukocytes, *T. gondii* needs to modulate cell adhesion, motility and TEM for efficient dissemination. Our data suggest that leukocyte ICAM-1 is targeted for these purposes.

We provide evidence that parasite effectors emanating from the dense granule and rhoptry secretory organelles, specifically GRA15 and TgWIP, are implicated in the enhanced TEM of parasitised DCs across endothelium (**Figure 5**). The data also provide a molecular framework for the previously observed parasite genotype-related differences in transmigration *in vitro* and dissemination in mice (45). Specifically, type II *T. gondii* strains relied to a higher extent than type I strains on transportation by DCs -*Trojan horse* mechanism- for systemic dissemination (26). In line with the present findings, type II parasites also induced a stronger upregulation of ICAM-1 by brain endothelial cells, compared with type I parasites (34). Further, we extend previous findings that TgWIP significantly impacts the motility and TEM of parasitised DCs (21) to polarised endothelium. However, TEM was not totally abolished in DCs infected with TgWIP-deficient parasites. Jointly, this indicated the implication of additional genotype-related unidentified effector(s). Here, we report that secreted GRA15 (type II) impacts transcriptional upregulation of ICAM-1 and TEM. Moreover, the GRA15-related elevated expression of ICAM-1 was accompanied by elevated adhesion and reduction of motility of type II-challenged DCs on endothelium, consistent with impacts on the cellular processes of crawling, arrest and adhesion that precede transmigration (3, 4). In contrast, DCs challenged with type I parasites, which lack a functional GRA15 (23), failed to upregulate ICAM-1 and, contrary to type II infected DCs, increased motility on endothelium, suggesting reduced adhesion and therefore a reduced capability to transmigrate. Indeed, because GRA15 acts on the transcription factor NFκB (23) and ICAM-1 transcription requires the binding of NFκB (8, 46), it is reasonable to assume that this mechanism is in play. Thus, the concerted action of GRA15 and TgWIP seem to mediate the major part of the TEM phenotype for the type II strains tested (ME49, PRU). However, in the type I strain (RH) that lacks a functional GRA15 (47, 48), TgWIP-deficiency reduced but did not totally abolish TEM. This indicates that additional identified and unidentified effector(s) may contribute to TEM, including Tg14-3-3 (49) and the polymorphic effector ROP17 (50). Moreover, a cautious interpretation of phenotypes using prototypic strains is needed. For example, the lack of a functional version of the polymorphic effector GRA15 cannot be generalized to all type I strains (47, 48). Further, it cannot be ruled out that additional polymorphic and non-polymorphic effectors impact the ICAM-1-related migratory processes in a strain-related fashion. For example, ROP18 in type I (RH) has been shown to inhibit the host NF-κB pathway (51), which could further explain the reduced expression of ICAM-1. Moreover, the traits need to be confirmed *in vivo* in future studies despite the challenges that tracking infected leukocytes represent (12, 17).

The implication of several putative parasite effectors in TEM is consonant with the complexity of the hypermigratory phenotype induced by *T. gondii* in infected mononuclear phagocytes, including DCs, monocytes, macrophages and microglia (15, 52, 53). Indeed, several central functions of leukocytes, such as cell locomotion, adhesion, chemotaxis and ability to transmigrate, are modulated by *T. gondii*. Previous work demonstrated that dramatic and rapid changes take place in parasitised phagocytes shortly after *T. gondii* infection, encompassing cytoskeletal remodelling, dissolution of adhesive podosomes, redistribution of integrins and modulation of extracellular matrix proteolysis (54–58). This implies the activation of several signalling pathways in the host cell (59), some of which are in response to the cellular environment and directly linked to integrin function (58, 60). Specifically, infected DCs transition between a high-speed integrin-independent amoeboid motility mode in extracellular matrix to an integrin-dependent motility mode on endothelium (24, 56). Here, we add the contribution of GRA15 to TEM through the induction of ICAM-1 expression in parasitized DCs. Of note, we previously found that treatment with LPS also upregulates ICAM-1 in DCs (24). However, LPS treatment does not induce hypermigration in DCs or other phagocytes (15, 61), indicating that ICAM-1 upregulation *per se* is not sufficient to induce the elevated TEM. We postulate that the concerted action of secreted polymorphic and non-polymorphic effectors mediates a migratory activation with modulated integrin function (24, 58, 60) and ICAM-1 expression in parasitised leukocytes, with an impact on the processes of TEM. Also, because the expression of cerebral endothelial E-selectin and VCAM-1 is modulated by *T. gondii* infection in mice (17), future studies need to determine their contribution to TEM.

The present work provides novel insights in how *T. gondii* orchestrates the modulation of host cell adhesion, motility and transmigration of parasitised leukocytes across polarised endothelium. While leukocytes facilitate the systemic dissemination of *T. gondii* (15, 16), their roles in mediating access to the brain parenchyma remain to be investigated (12). The infection of leukocytes by *T. gondii* may serve dual effects. The host leukocyte represents a replicative niche where the parasite is protected from immune attack in the PV, while transportation of intracellularly located parasites in migratory leukocytes facilitates dissemination.

## Data availability statement

The original contributions presented in the study are included in the article/**supplementary material**. Further inquiries can be directed to the corresponding author.

## Ethics statement

The animal study was reviewed and approved by The Regional Animal Research Ethical Board, Stockholm, Sweden.

## Author contributions

ER and AH performed experiments and analysed the data. JS provided valuable reagents. All authors contributed to the writing of this manuscript.

## Funding

This work was funded by the Swedish Research Council (Vetenskapsrådet, 2018–02411) and the Olle Engkvist Foundation (193–609).

## Acknowledgments

We thank Dr. Manuel Varas-Godoy, San Sebastian University, Chile, for expert advice on lentiviral transduction.

## Conflict of interest

The authors declare that the research was conducted in the absence of any commercial or financial relationships that could be construed as a potential conflict of interest.

## Publisher’s note

All claims expressed in this article are solely those of the authors and do not necessarily represent those of their affiliated organizations, or those of the publisher, the editors and the reviewers. Any product that may be evaluated in this article, or claim that may be made by its manufacturer, is not guaranteed or endorsed by the publisher.

## Supplementary material

Supplementary Figure 1 Infection frequency and assessments of polarisation and barrier integrity for transmigration assays. **(A)** Infection frequency of DCs challenged with type I or type I *T. gondii* at MOI 2 for 4 h. **(B)** TEER values of bEnd.3 cells relative to TEER values at initiation of the assay (100%), in . **(C)** Permeability of bEnd.3 cell monolayers to FITC-dextran (3 kDa) following TEM in . **(D)** Relative expression of ICAM-1 (CD54) at 24 h post-challenge of CD11c^+^ cells with type II (PRU) *T. gondii* tachyzoites (WT or ΔMYR1, MOI 1), assessed by flow cytometry. Mean fluorescence intensity (MFI) was related to that of unchallenged CD11c^+^ in complete medium (CM, normalised to 1). Bar graphs represent the mean ± s.e.m of 3-4 independent experiments. ns: non-significant, by one-way ANOVA, Sidak’s *post-hoc* test.

Supplementary Figure 2 Transduction of primary DCs. Representative micrographs of mock-treated DCs (Mock) and eGFP-expressing DCs transduced with shLuc or shICAM-1, as described in materials and methods. Transduction efficiency was consistently 30-40% based on eGFP expression. Scale bar = 100µm.
