# Supplementary figures and images for: Toxoplasma effector-induced ICAM-1 expression by infected dendritic cells potentiates transmigration across polarised endothelium
Source: Front Immunol. 2022 Aug 3;13:950914. doi: 10.3389/fimmu.2022.950914 (PMC9381734; doi:10.3389/fimmu.2022.950914)

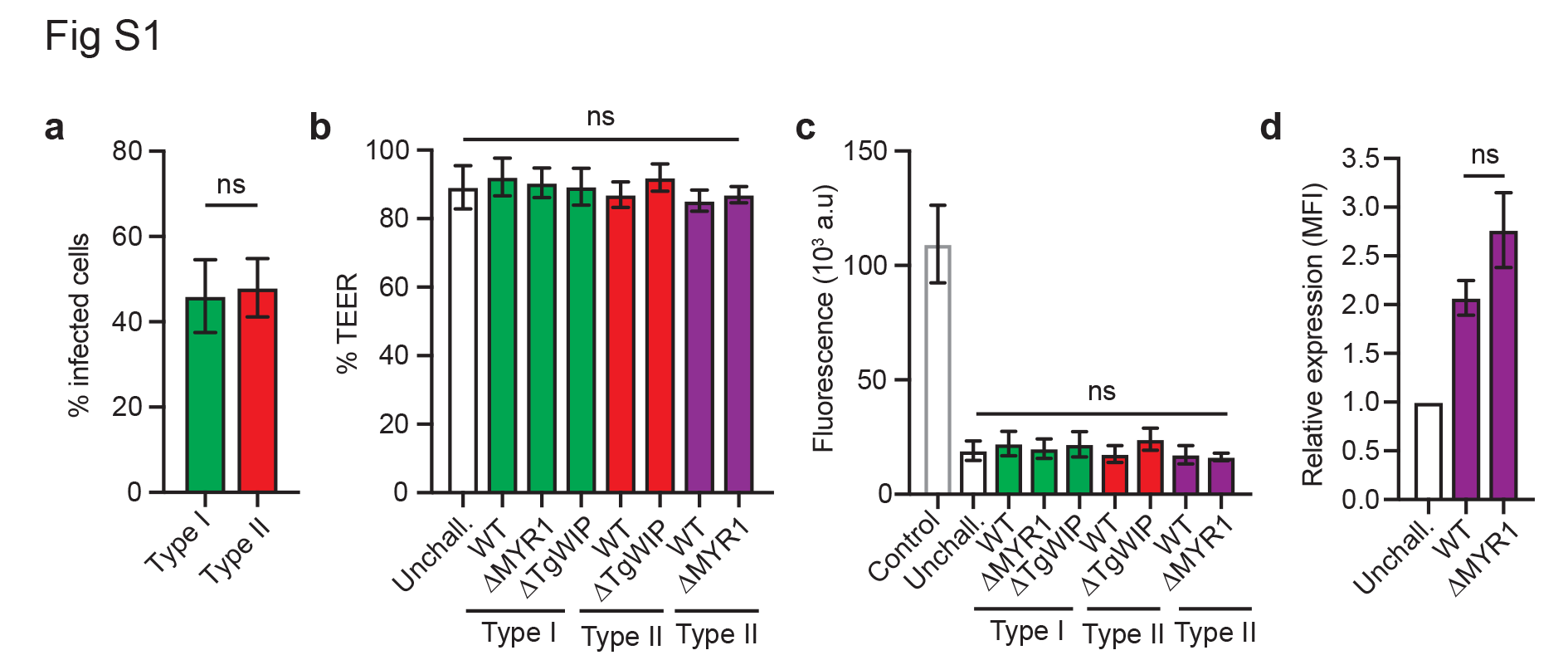

Supplement: Supplementary Figure 1 — Infection frequency and assessments of polarisation and barrier integrity for transmigration assays. (A) Infection frequency of DCs challenged with type I or type I T. gondii at MOI 2 for 4 h. (B) TEER values of bEnd.3 cells relative to TEER values at initiation of the assay (100%), in . (C) Permeability of bEnd.3 cell monolayers to FITC-dextran (3 kDa) following TEM in . (D) Relative expression of ICAM-1 (CD54) at 24 h post-challenge of CD11c+ cells with type II (PRU) T. gondii tachyzoites (WT or ΔMYR1, MOI 1), assessed by flow cytometry. Mean fluorescence intensity (MFI) was related to that of unchallenged CD11c+ in complete medium (CM, normalised to 1). Bar graphs represent the mean ± s.e.m of 3-4 independent experiments. ns: non-significant, by one-way ANOVA, Sidak’s post-hoc test. [file Image_1.tif]

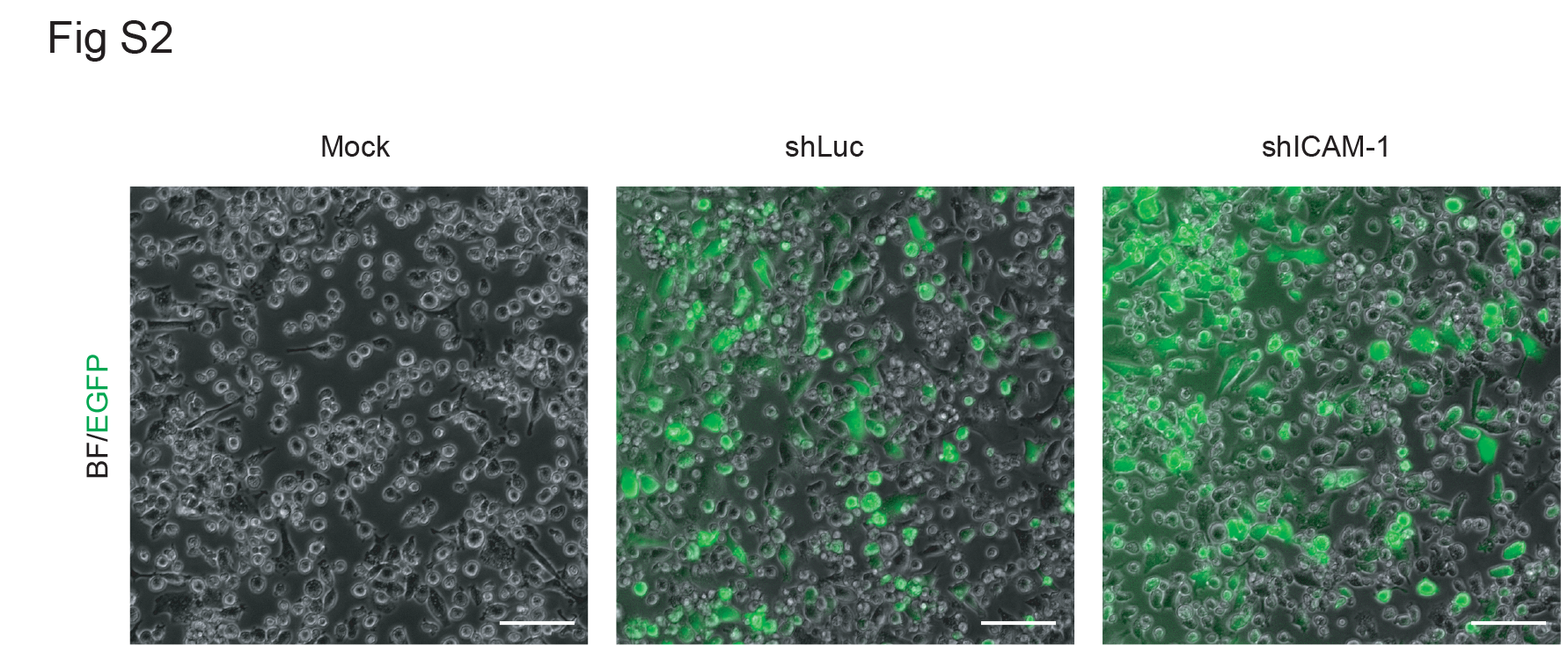

Supplement: Supplementary Figure 2 — Transduction of primary DCs. Representative micrographs of mock-treated DCs (Mock) and eGFP-expressing DCs transduced with shLuc or shICAM-1, as described in materials and methods. Transduction efficiency was consistently 30-40% based on eGFP expression. Scale bar = 100µm. [file Image_2.tif]
