# Supplementary material for: Toxoplasma effector-induced ICAM-1 expression by infected dendritic cells potentiates transmigration across polarised endothelium
Source: Front Immunol. 2022 Aug 3;13:950914. doi: 10.3389/fimmu.2022.950914 (PMC9381734; doi:10.3389/fimmu.2022.950914)
Supplement: Supplementary file 3 [file Table_1.docx]

**Table S1. *T. gondii* lines**

| *Strain name* | *reference* |
| --- | --- |
| *T. gondii* ME49-PTG GFPS65T | [1] |
| *T. gondii* ME49 RFP | [2] |
| *T. gondii* RH1-1 cLuc GFP | [3] |
| *T. gondii* RH ΔMYR1 cLuc GFP | [4] |
| *T. gondii* PRUΔku80 | [5] |
| *T. gondii* PRUΔMYR1 | [5] |
| *T. gondii* RH ΔTgWIP GFP | [6] |
| *T. gondii* ME49 ΔTgWIP GFP | [6] |
| *T. gondii* PRU PA7 GFP | [7] |
| *T. gondii* PRU PA7 GFP ΔGRA15 | [7] |

1. Kim K, Eaton MS, Schubert W, Wu S, Tang J. Optimized expression of green flourescent protein in *Toxoplasma gondii* using thermostable green flourescent protein mutants. Mol Biochem Parasitol. 2001;113:309-13.

2. Wang Y, Cirelli KM, Barros PDC, Sangare LO, Butty V, Hassan MA, et al. Three Toxoplasma gondii Dense Granule Proteins Are Required for Induction of Lewis Rat Macrophage Pyroptosis. mBio. 2019;10(1). Epub 2019/01/10. doi: 10.1128/mBio.02388-18. PubMed PMID: 30622189; PubMed Central PMCID: PMCPMC6325250.

3. Boyle JP, Saeij JP, Boothroyd JC. Toxoplasma gondii: inconsistent dissemination patterns following oral infection in mice. Experimental parasitology. 2007;116(3):302-5. Epub 2007/03/06. doi: 10.1016/j.exppara.2007.01.010. PubMed PMID: 17335814.

4. Wang Y, Sangare LO, Paredes-Santos TC, Hassan MA, Krishnamurthy S, Furuta AM, et al. Genome-wide screens identify Toxoplasma gondii determinants of parasite fitness in IFNgamma-activated murine macrophages. Nature communications. 2020;11(1):5258. Epub 2020/10/18. doi: 10.1038/s41467-020-18991-8. PubMed PMID: 33067458; PubMed Central PMCID: PMCPMC7567896.

5. Jensen KD, Wang Y, Wojno ED, Shastri AJ, Hu K, Cornel L, et al. Toxoplasma polymorphic effectors determine macrophage polarization and intestinal inflammation. Cell host & microbe. 2011;9(6):472-83. Epub 2011/06/15. doi: 10.1016/j.chom.2011.04.015. PubMed PMID: 21669396; PubMed Central PMCID: PMCPMC3131154.

6. Sangare LO, Olafsson EB, Wang Y, Yang N, Julien L, Camejo A, et al. In Vivo CRISPR Screen Identifies TgWIP as a Toxoplasma Modulator of Dendritic Cell Migration. Cell host & microbe. 2019;26(4):478-92 e8. Epub 2019/10/11. doi: 10.1016/j.chom.2019.09.008. PubMed PMID: 31600500.

7. Rosowski EE, Lu D, Julien L, Rodda L, Gaiser RA, Jensen KD, et al. Strain-specific activation of the NF-kappaB pathway by GRA15, a novel Toxoplasma gondii dense granule protein. The Journal of experimental medicine. 2011;208(1):195-212. Epub 2011/01/05. doi: 10.1084/jem.20100717. PubMed PMID: 21199955; PubMed Central PMCID: PMCPMC3023140.

**Table S2. Sequences for shRNAs**

| **Target** | | **Sequence (5’ to 3’)** |
| --- | --- | --- |
| shLuc: | TGTTCTCCGAACGTGTCACGTTTCAAGAGAACGTGACACGTTCGGAGAACTTTTTTC | |
|  |  | |
| shIcam1: | CCGGACGCTGACTTCATTCTCTATTCTCGAGAATAGAGAATGAAGTCAGCGTTTTTTG | |

**Table S3. Sequences for qPCR primers**

| **Target** | **Primer pair sequence (5’ to 3’)** |
| --- | --- |
| *Gapdh* | Fd: TGACCTCAACTACATGGTCTACA  Rv: CTTCCCATTCTCGGCCTTG |
| *Icam1* | Fd: CAATTTCTCATGCCGCACAG  Rv: CTGGAAGATCGAAAGTCCGG |
